# Stroke risk following bee and wasp stings: a systematic review of ischemic and hemorrhagic events

**DOI:** 10.3389/ftox.2025.1632308

**Published:** 2025-08-25

**Authors:** Jorge Vasconez-Gonzalez, Juan S. Izquierdo-Condoy, Karen Delgado-Moreira, María de Lourdes Noboa-Lasso, Esteban Gamez-Rivera, Camila Salazar-Santoliva, María Belén Lopez-Molina, Esteban Ortiz-Prado

**Affiliations:** ^1^ One Health Research Group, Faculty of Health Science, Universidad de Las Americas, Quito, Ecuador; ^2^ Program in Occupational Safety and Health, The University of Porto, Porto, Portugal

**Keywords:** bee stings, wasp stings, neurovascular complications, venom-induced stroke, cerebrovascular events, insect envenomation, neurological toxicity

## Abstract

**Background:**

Each year, approximately 100 million cases of bee and wasp stings are re-ported globally, with the majority resulting in mild reactions. However, in rarer instances, these stings can lead to severe and potentially fatal outcomes, including ischemic or hemorrhagic cerebral events. This article aims to synthesize and analyze the current evidence on the association between bee and wasp stings and the occurrence of ischemic and hemorrhagic strokes.

**Methodology:**

A systematic review was conducted in accordance with PRISMA guidelines. Searches were performed in PubMed, Scopus, and Scielo databases, including studies published in English and Spanish without time restrictions. Studies that met the inclusion criteria, specifically focusing on “bee sting” or “wasp sting” and “stroke” or “cerebrovascular disease” in humans, were included.

**Results:**

Out of the 83 articles initially identified, 28 met the inclusion criteria and were included in this systematic review, documenting a total of 29 cases of stroke associated with bee or wasp stings. The distribution of cases was nearly equal between bee and wasp stings. Ischemic stroke emerged as the most commonly reported type, with clinical manifestations primarily affecting the nervous system. Common symptoms included hemiparesis or hemiplegia, hypertension, dysarthria or aphasia, and loss of consciousness or syncope. This pattern underscores the significant neurological and systemic impact of envenomation, which, while rare, can lead to severe and potentially life-threatening complications.

**Conclusion:**

While cerebrovascular events like ischemic and hemorrhagic strokes following bee or wasp stings are rare, the risk is significant and can be life-changing. The impact of a stroke extends beyond immediate symptoms, affecting long-term quality of life. Therefore, it is crucial that healthcare facilities establish protocols to recognize and manage these rare but severe complications. Further research is needed to better understand and mitigate this risk.

## 1 Introduction

The interconnected relationship between humans, animals, and the environment has increased human exposure to a wide range of animal species, including insects. These insects, which are distributed globally, interact with humans in various environments and habitats across the planet. In this context, wasps (*Vespidae*), bees (*Apidae*), and hornets (*Vespidae*), which belong to the order *Hymenoptera* (Wani et al., 2014), are responsible for a significant number of stings worldwide and account for the majority of hypersensitivity reactions to insect stings (Diaz, 2009). The burden of these reactions is difficult to estimate; however, some reports suggest that they account for nearly 100 million cases per year, ranging from local reactions to fatalities caused by anaphylactic shock (Feás, 2021). Although stings are more common among young adult males engaging in outdoor activities, every age group is vulnerable (Smallheer, 2013). Allergic reactions are the most common medical consequence, affecting up to one-third of victims, including those with no prior history of allergies (Diaz, 2009).

The sting mechanism and type of venom exhibit chemical, molecular, and other similarities between bees and wasps (Habermann, 2013). Bees inflict a single sting, and their stingers detach, whereas wasps can inflict multiple stings because their stingers have fewer barbs and can be easily removed without detaching (Diaz, 2009). The venoms of bees and wasps differ in composition; for example, bee venom contains melittin, while wasp venom includes a protein known as antigen 5, which is found in the venom of hornets, yellowjackets, and other wasps, and is a significant allergen for individuals sensitive to insect stings (Kochoumian and Lam, 1987). Both venoms also contain hyaluronidases and phospholipases, which produce various effects on the human body (Diaz, 2009; Wani et al., 2014). The most common effects are those associated with the cardinal signs of inflammation, including edema, erythema, burning, pruritus, urticaria, and angioedema, which usually resolve within 24 h (Gupta, 2020). More severe, though less common, complications have been described, including anaphylactic shock and hypotension, as well as myocardial infarction, acute renal failure, pulmonary hemorrhage, rhabdomyolysis, acute hemorrhagic pancreatitis, atrial fibrillation, seizures, disseminated intravascular coagulation (DIC), intracranial hemorrhages, and cerebral infarctions (Temizoz et al., 2009; Gupta, 2020). An insightful classification by Müller in 1990 categorized reactions to Hymenoptera stings into four types: local, large local, systemic (grades I to IV), and unusual delayed reactions. Neurological complications, including strokes, fall into the category of unusual delayed reactions (Müller, 1990).

Among the rarest yet most severe consequences of bee and wasp envenomation are ischemic or hemorrhagic strokes (Temizoz et al., 2009; Kulhari et al., 2016). Although vascular complications following bee or wasp stings are rare, they should not be overlooked, as their consequences can create a significant burden and have a greater individual impact than other symptoms. The mechanisms by which bee and wasp stings can cause strokes are not fully understood, but several hypotheses have been proposed (Kulhari et al., 2016; Gupta, 2020; Kabra et al., 2022). These mechanisms include hyperactivity of the immune system, global cerebral hypoperfusion, retrograde stimulation of the superior cervical ganglion, disseminated intravascular coagulation, and vasoconstriction, which can lead to cellular hypoxia, ischemia, and subsequent necrosis of neural tissue (Yang et al., 2022). This is related to the action of venom components such as histamine, thromboxane, leukotrienes, and other vasoactive and inflammatory mediators that induce platelet aggregation and vasoconstriction (Mahale et al., 2016; Dalugama and Gawarammana, 2018; Gupta, 2020).

Despite the limited understanding of the mechanisms due to the rarity of these events, their widespread geographical distribution, and the lack of extensive research, we believe it is crucial to report, summarize, and consolidate the information on this topic. The consequences can be devastating not only for the patient but also for their family and the healthcare system. Therefore, this article aims to integrate and analyze information related to ischemic and hemorrhagic strokes associated with bee and wasp stings.

## 2 Methodology

### 2.1 Research question

Can bee and wasp stings be a contributing factor in the onset of a stroke, whether ischemic or hemorrhagic, and what are the underlying mechanisms that may link these stings to cerebrovascular events?

### 2.2 Study desing

We conducted a systematic review of case reports on stroke triggered by bee and wasp stings, following the PRISMA (Preferred Reporting Items for Systematic Reviews and Meta-Analyses) methodology, a recommended guideline for conducting systematic reviews and meta-analyses (Supplementary Table S1). The protocol for this research was registered in Prospero under the code: CRD42024572815.

### 2.3 Search strategies

A bibliographic search was conducted in English and Spanish, with no time limit. The databases used were PubMed, Scopus, and Scielo. Additionally, an analysis of the reference lists of the selected articles was performed to access relevant studies not found through the databases used. The following syntax was used for the bibliographic search with indexed terms, keywords, and Boolean operators: (“bee sting” OR “wasp sting”) AND (“stroke” OR “cerebrovascular disease” OR “ischemic stroke” OR “hemorrhagic stroke”) in the title or abstract.

### 2.4 Selection criteria

#### 2.4.1 Inclusion criteria

- Clinical Studies (those studies involving humans)
- Studies in which the presence of a cerebrovascular event is confirmed by imaging tests

#### 2.4.2 Exclusion criterion

- Studies in which the presence of a cerebrovascular event is not confirmed by imaging tests
- Studies performed in animals
- Studies that evaluate the stings of other insects
- Studies that evaluate neurological complications of bee and wasp stings other than stroke

### 2.5 Study selection

**FIGURE 1:**
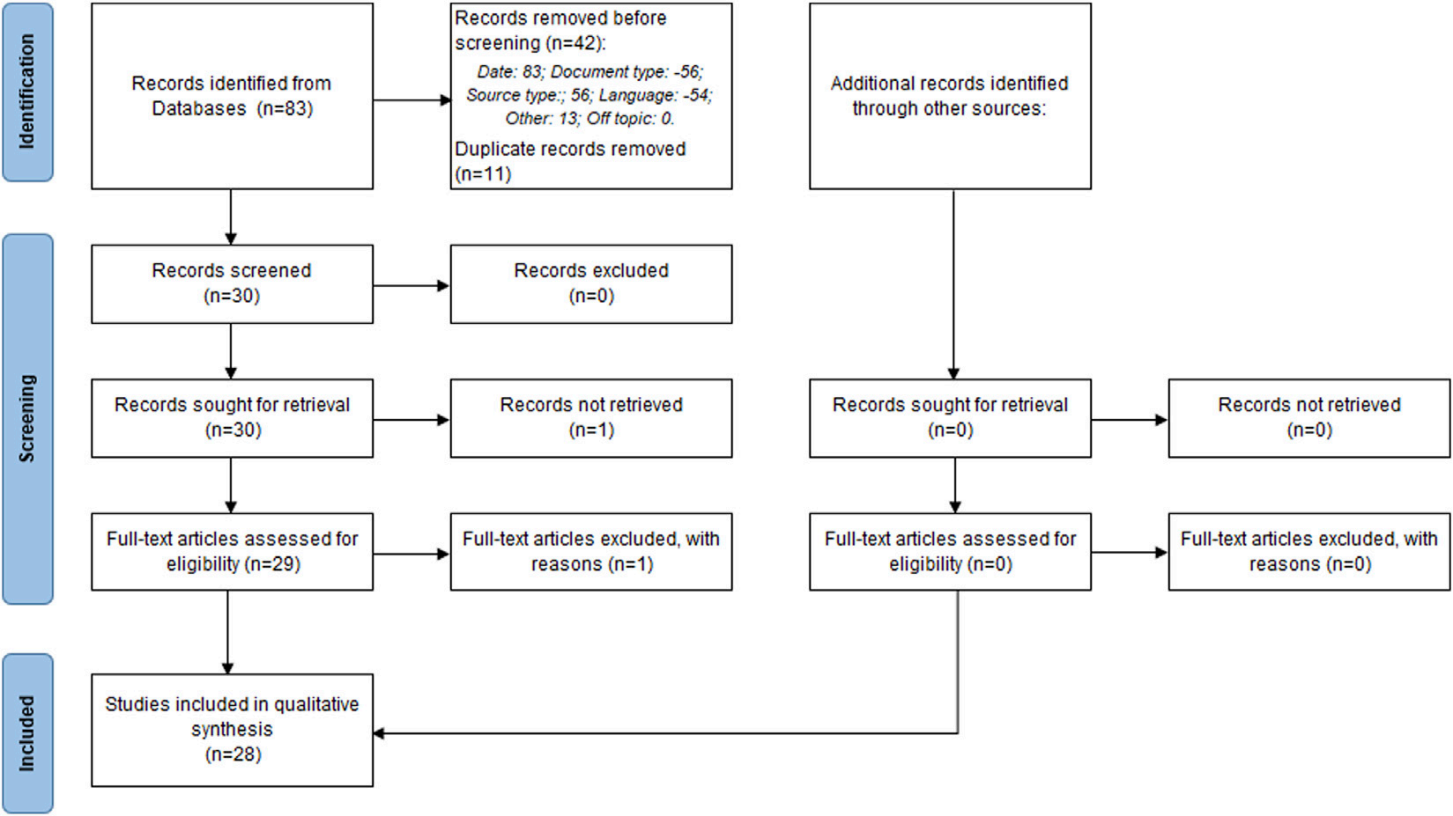
PRISMA flowchart illustrating the study selection process for this systematic review, detailing the number of studies screened, assessed for eligibility, and included in the review, with reasons for exclusions at each stage.

The initial bibliographic search identified 83 articles, of which 42 were eliminated in the first phase prior to screening, and 11 were eliminated due to duplication, resulting in 30 eligible studies. During the screening, 2 studies were eliminated, leaving a total of 28 studies included in this review (Figure 1).

### 2.6 Bias Assessment

To minimize the risk of bias, the data extraction process was performed independently by JEV and KDM at different times. Discrepancies in data collection from a primary study were resolved through discussion and consensus.

### 2.7 Data synthesis

We performed a comprehensive review of all manuscripts that met our inclusion criteria. The quality of the studies was assessed using the JBI critical appraisal checklist for case reports. Studies were categorized as low, moderate, or high quality based on this scale. Finally, information from the manuscripts related to the research question was organized and synthesized into tables.

## 3 Results

A total of 28 studies were included in this systematic review. Following the quality assessment of the studies using the JBI Critical Appraisal Checklist for Case Reports, 25 were rated as high quality with a score of 8, and 3 as moderate quality with a score of 7 (Supplementary Table S2). Among the 29 stroke cases identified, 14 (48.3%) were due to bee stings and 15 (51.7%) were due to wasp stings. The mean age of the patients was 45.79 years (SD 15.6). Almost all cases were recorded in males, with 28 (96.6%) of cases. Ischemic stroke was the most frequent type, occurring in 26 (89.7%) cases.

Clinical manifestations associated with these strokes varied but predominantly affected the nervous system, followed by respiratory complications. The most common symptoms included hemiparesis, aphasia, and facial nerve palsy. Detailed characteristics and clinical findings of the patients are summarized in Table 1.

**TABLE 1:** Main characteristics and clinical findings of patients who experienced stroke following bee or wasp stings. This table summarizes the demographic details, symptoms, imaging findings, and stroke types observed in the 29 cases included in this review. Most cases involved male patients, with ischemic strokes being the predominant type. The table also provides specific clinical presentations and imaging results that illustrate the severity and nature of the cerebrovascular events triggered by envenomation.

Author	Sex	Age (years)	Sting	Symptoms	Imaging findings	Stroke type
Riggs et al. (1994)	Male	52	Wasp	-Dizzy -Respiratory arrest -Syncope -Hypotension -Slurred speech -Left hemiparesis	CT: Bilateral cerebral edema, three focal ischemic lesions, tow in the right centrum semiovale, and one in the right temporal lobe	Ischemic
Guzel et al. (2016)	Male	59	Bee	-Hypertension -Decreased consciousness with stupor -Left central facial paralysis -Left hemiplegia	MRI: Acute infarction extended from the right frontotemporal region to parietal region	Ischemic
Kabra et al. (2022)	Male	49	Bee	-Hypertension -Tachycardia -Tachypnea -Generalized tonicclonic seizure -Right hemiparesis	MRI: Hyperintensities in the left corona radiata	Ischemic
Ramlackhansingh and Seecheran (2020)	Male	70	Bee	-Right hemiparesis -Dysphasia	MRI: Ischemic stroke in the left hemisphere	Ischemic
Masaraddi et al. (2021)	Male	45	Bee	-Right hemiplegia -Aphasia -Hypertension	MRI and TC: Hyperacute infarcts affecting the frontoparietal, temporal lobes, and basal ganglia on the left side	Ischemic
Vidhate et al. (2011)	Male	8	Wasp	-Ophthalmoplegia -Ptosis -Left hemiplegia -Proptosis -Altered sensorium - Facial nerve palsy - Reduced range of eye movements -Hyperreflexia	CT: No hemorrhagic infarcts in left frontoparietal and bilateral subcortical regions	Ischemic
Alvis- Miranda et al. (2014)	Male	74	Bee	-Paresthesia -Language alteration -Right hemiparesis -Hypertension - Murmur in the left common carotid artery -Facial paralysis -Hyperactive reflexes	CT: Left frontal hypodensity suggestive of brain regional infarction	Ischemic
Yang et al. (2022)	Male	63	Bee	-Dysarthria -Bulbar weakness -Right hemiparesis	CT: Acute infarcts in the left parieto-occipital region and left thalamus	Ischemic
An et al. (2014)	Male	50	Bee	-Syncope -Hypertension -Hyperkinetic movements	MRI: Right temporal lobe infarction	Ischemic
Kulhari et al. (2016)	Male	44	Wasp	-Left hemiparesis -Facial weakness -Dysarthria -Hypertension	MRI: Middle cerebral artery territory ischemic stroke	Ischemic
Nittner-Marszalska et al. (2022)	Male	44	Wasp	-Lost consciousness -Stiff neck -Facial muscles’ dyskinesia -Aphasia -Facial nerve palsy -Right hemiparesis -Hyperreflexia	MRI: Chronic hypoxic-ischemic lesions of the head and the body of the caudate nucleus and putamen bilaterally, further chronic ischemic changes within the cortex and subcortical white matter of the left parietal lobe with segmental cortical atrophy	Ischemic
	Male	24	Wasp	-Lost consciousness -Left hemiparesis	MRI: Acute ischemic changes within the cortex of the postcentral gyrus and subcortical white matter of the right parietal lobe and right frontal lobe	Ischemic
Dalugama and Gawarammana (2018)	Male	69	Wasp	-Slurring of speech -Deviation of mouth to the left side -Right Hemiparesis -Hypertension -Aphasia -Facial nerve palsy	MRI: Acute infarction in the left posterior frontal white matter	Ischemic
Wani et al. (2014)	Male	40	Wasp	-Deterioration in consciousness -Vomiting -Incontinence of urine -Fever -Hypertension	CT: Hyperintense lesion in left parietooccipital region and right cerebellum	Ischemic
Rajendiran et al. (2012)	Male	25	Bee	-Left sided monoparesis -Transient visual loss	CT: Right frontal hypodensities with squashing of ipsilateral ventricles with hypodensities over both occipital lobes MRI: Anterior infarct right frontoparietal region, right occipital region	Ischemic
Temizoz et al. (2009)	Male	60	Bee	-Hypertension -Left hemiplegia -Dysarthria	MRI: Ischemic changes in the frontal lobes, right temporoparietal area, and bilateral centrum semiovale	Ischemic
Karri et al. (2021)	Male	40	Wasp	-Tonic-clonic seizures - Frothing -Urinary incontinence -Hypotension -Tachypnea -Tachycardia	MRI: Massive infarct in the anterior and middle cerebral artery regions with right internal carotid artery thrombosis	Ischemic
Sachdev et al. (2002)	Male	40	Wasp	-Left hemiparesis -Lurring of speech -Deviation of angle of mouth to the left side -Dysarthria -Hypertension -Hyperreflexia -Facial palsy	CT and MRI: Non-hemorrhagic infarct in the right ventral pons and the posterior superior part of right half of cerebellum	Ischemic
Riggs et al. (1993)	Male	38	Wasp	-Right hemiparesis -Luring of speech	CT and MRI: Ischemic infraction in the distribution of left middle cerebral artery	Ischemic
Priyadarshi et al. (2024)	Male	40	Wasp	-Respiratory distress -Somnolence -Disorientation -Tachycardia -Tachypnea -Hypertension	MRI: Hyperintensity involving bilateral paramedian thalami and rostral midbrain	Ischemic
Bilir et al. (2013)	Male	35	Bee	-Change in consciousness -Dyspnea -Respiratory distress -Tachycardia -Tachypnea	MRI: Left middle cerebral artery infarction	Ischemic
Bong et al. (2017)	Male	52	Wasp	-Decreased consciousness -Dyspnea -Hypotension -Aphasia -Right hemiparesis	MRI: High-intensity signals in parts of the left basal ganglia and cerebral cortex	Ischemic
Ciron et al. (2015)	Male	46	Wasp	-Hypotension -Diplopia -Mild cerebellar ataxia -Meningism	MRI: Multiple small cerebral hemorrhages associated with a right thalamic infarct; bilateral occipital subarachnoid hemorrhage	Hemorrhagic
Elavarasi et al. (2020)	Male	41	Bee	-Lost consciousness -Left hemiparesis -Dysarthria	CT: Hypodensity in the right middle cerebral artery territory suggesting infarction	Ischemic
Evcen et al. (2023)	Male	48	Bee	-Dyspnea -Syncope - Facial paralysis Hypoesthesia	MRI: Acute lacunar infarct in the pons	Ischemic
Halasah (2013)	Female	75	Wasp	-Deviation of mouth to the left side -Aphasia -Weakness -Facial palsy	CT: Ischemic infraction in the distribution of left middle cerebral artery	Ischemic
Jain et al. (2012)	Male	70	Bee	-Altered sensorium -Hypertension -Aphasia -Right hemiparesis	CT: Multiple acute infarcts and multiple lacunar infarcts in bilateral ganglio capsular regions involving the left caudate nucleus, right lentiform nucleus and bilateral external capsule MRI: Subacute hemorrhagic infarcts in the left parietooccipital region with old lacunar infarcts in the bilateral external capsule	Hemorrhagic
Reddy et al. (2023)	Male	55	Wasp	-Weakness -Hyperreflexia	CT: Large intraparenchymal hemorrhage involving the right ganglio-thalamo-capsular region extending into the intraventricular, subarachnoid hemorrhage in bilateral fronto-parietal sulcal spaces and along the falx cerebri	Hemorrhagic
Viswanathan et al. (2012)	Male	59	Bee	-Slurred speech -Left hemiplegia -Facial nerve palsy -Left conjugate gaze palsy -Depressed gag reflex	CT: Narrowing of right Middle cerebral artery territory gyri MRI: Diffuse altered signal intensity along perisylvian, peri-insular, and parietal cortices	Ischemic

CT, computed tomography; MRI, magnetic resonance imaging.

**FIGURE 2:**
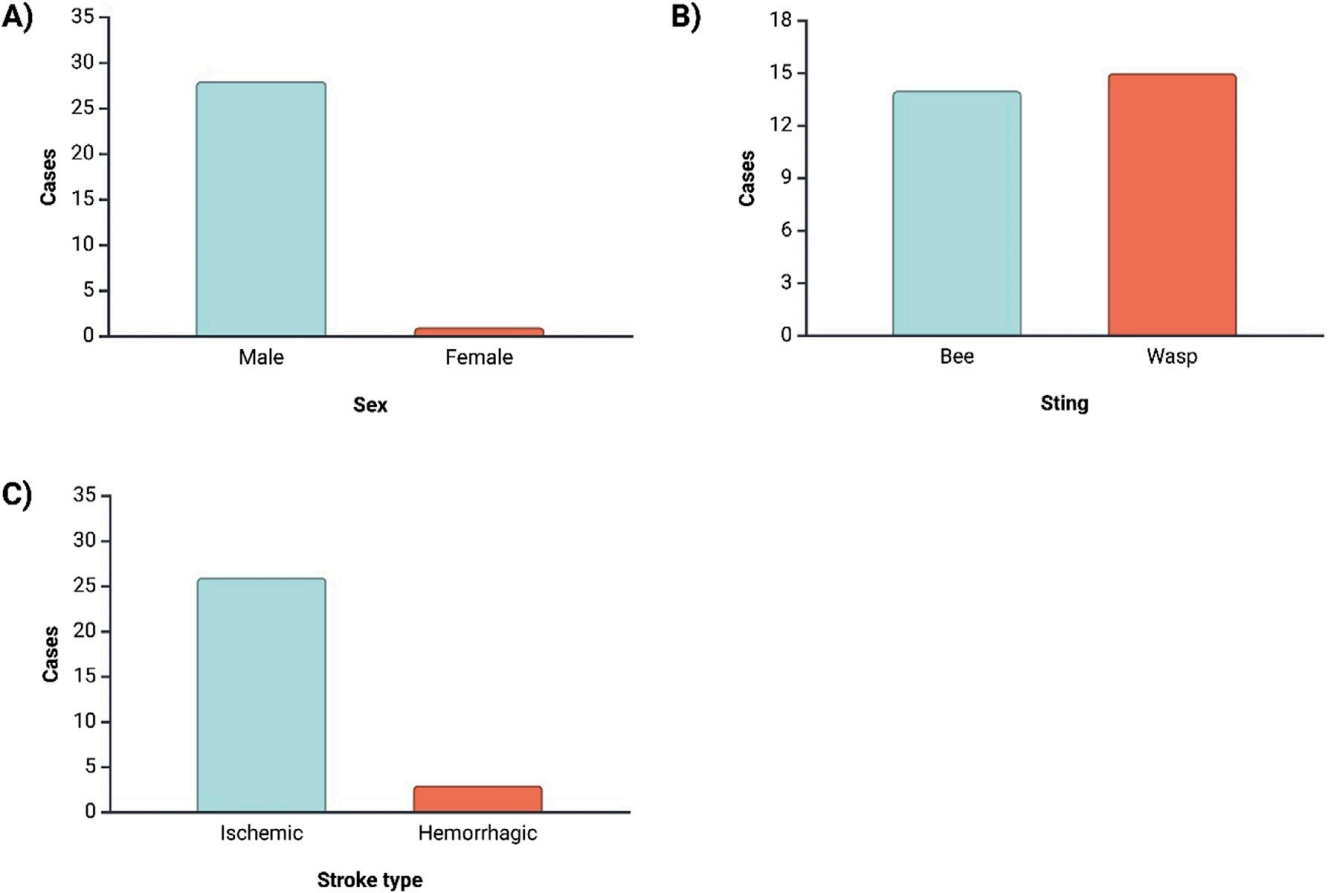
This image presents the distribution of key demographic and clinical characteristics among the patients who experienced stroke as a result of bee or wasp stings. Panel **(A)** shows that a significant majority of stroke cases occurred in males (96.6%), highlighting a potential gender predisposition. Panel **(B)** illustrates that the distribution of stroke cases is nearly equal between bee and wasp stings, with a slight predominance of wasp stings (51.7%). Panel **(C)** indicates that ischemic strokes were the most frequent type, occurring in 89.7% of the cases, compared to hemorrhagic strokes.

Following the analysis of clinical data, it is essential to examine both the demographic and clinical distribution of the cases included in this review. The demographics offer valuable insights into factors such as age, gender, and the type of stroke associated with bee or wasp stings, which can influence the likelihood of stroke following envenomation, as presented in Figure 2.

### 3.1 Clinical manifestations

The central nervous system manifestations was the most affected, with clinical manifestations including right hemiparesis, facial nerve palsy, speech and language disorders, alterations in consciousness, aphasia, left hemiparesis, dysarthria, left hemiplegia, hyperreflexia, syncope, mouth deviation to the left side, and seizures. The respiratory system also exhibited significant involvement, with symptoms such as tachypnea, dyspnea, and respiratory distress, while the cardiovascular system presented with hypertension, tachycardia, and a murmur in the left common carotid artery (Table 1) (Riggs et al., 1993; 1994; Sachdev et al., 2002; Temizoz et al., 2009; Vidhate et al., 2011; Jain et al., 2012; Rajendiran et al., 2012; Viswanathan et al., 2012; Bilir et al., 2013; Halasah, 2013; Alvis- Miranda et al., 2014; An et al., 2014; Wani et al., 2014; Ciron et al., 2015; Guzel et al., 2016; Kulhari et al., 2016; Bong et al., 2017; Dalugama and Gawarammana, 2018; Elavarasi et al., 2020; Ramlackhansingh and Seecheran, 2020; Karri et al., 2021; Masaraddi et al., 2021; Kabra et al., 2022; Nittner-Marszalska et al., 2022; Yang et al., 2022; Evcen et al., 2023; Reddy et al., 2023; Priyadarshi et al., 2024).

**FIGURE 3:**
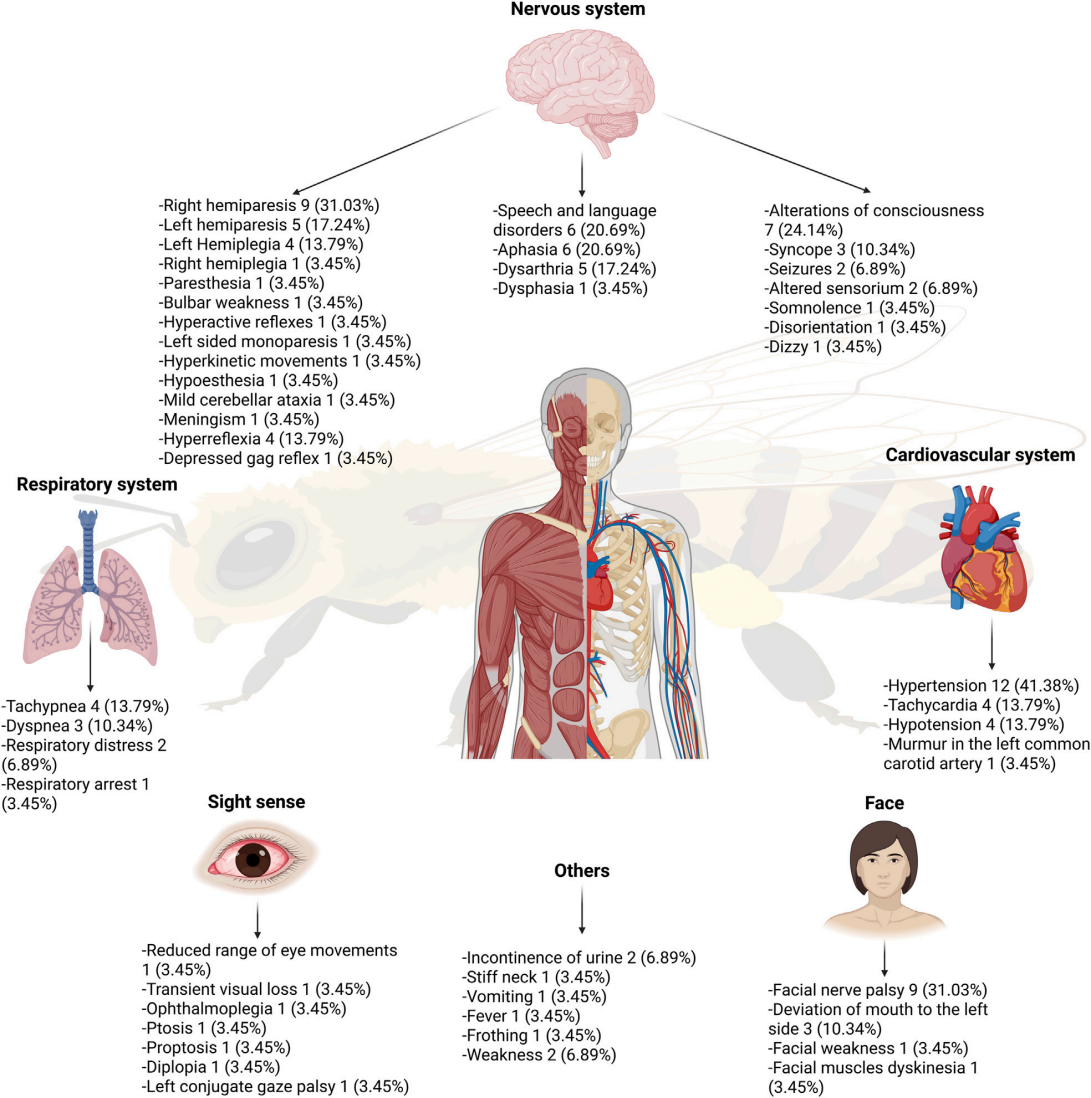
Clinical manifestations developed after bee and wasp stings.

In terms of frequency, hypertension was the most common symptom, occurring in 41.38% (n = 12) of cases (in comparison, hypotension was reported in 13.79% (n = 4) of the patients), followed by right hemiparesis and facial nerve palsy, each observed in 31.03% (n = 9) of cases. Alterations in consciousness were reported in 24.14% (n = 7) of cases, Speech and language disorders were reported and aphasia were reported in 20.69% (n = 6) (Figure 3). Less frequent symptoms included a reduced range of eye movements, paresthesia, disorientation, diplopia, dizziness, mild cerebellar ataxia, meningism, and ophthalmoplegia.

There is no clearly defined timeframe for stroke development; published case reports describe a wide range in time to onset, from less than 1 hour to as much as 48 h (Riggs et al., 1993; 1994; Sachdev et al., 2002; Jain et al., 2012; Viswanathan et al., 2012; Bilir et al., 2013; Halasah, 2013; Alvis- Miranda et al., 2014; An et al., 2014; Wani et al., 2014; Ciron et al., 2015; Guzel et al., 2016; Kulhari et al., 2016; Bong et al., 2017; Dalugama and Gawarammana, 2018; Elavarasi et al., 2020; Ramlackhansingh and Seecheran, 2020; Karri et al., 2021; Masaraddi et al., 2021; Kabra et al., 2022; Nittner-Marszalska et al., 2022; Priyadarshi et al., 2024).

### 3.2 Imaging findings

The primary imaging modalities used were computed tomography (CT) and magnetic resonance imaging (MRI). For hemorrhagic stroke, CT findings revealed intraparenchymal hemorrhage in the right gangliothalamocapsular region extending into the intraventricular space, subarachnoid hemorrhage in bilateral frontoparietal sulcal spaces and along the falx cerebri, as well as lacunar infarcts in bilateral gangliocapsular regions (Jain et al., 2012; Reddy et al., 2023). MRI identified small cerebral hemorrhages with right thalamic infarcts, bilateral occipital subarachnoid hemorrhage, and subacute hemorrhagic infarcts in the left parietooccipital region (Table 1) (Jain et al., 2012; Ciron et al., 2015).

For ischemic stroke, CT findings included cerebral edema, ischemic lesions in the right centrum semiovale, bilateral temporal lobes, left thalamus, and infarcts within the distribution of the middle cerebral arteries. Other findings included infarcts in the left frontoparietal region, bilateral subcortical regions, left parietooccipital region, right ventral pons, posterior superior part of the right cerebellum, bilateral frontal hypodensity, occipital lobe hypodensity, and hyperintense lesions in the right cerebellum (Table 1) (Riggs et al., 1993; 1994; Sachdev et al., 2002; Vidhate et al., 2011; Rajendiran et al., 2012; Viswanathan et al., 2012; Halasah, 2013; Alvis- Miranda et al., 2014; Wani et al., 2014; Elavarasi et al., 2020; Masaraddi et al., 2021; Yang et al., 2022). MRI findings further revealed right internal carotid artery thrombosis, diffuse altered signal intensity in the perisylvian, periinsular, and parietal cortices, as well as high-intensity signals in the left basal ganglia, cerebral cortex, left corona radiata, bilateral paramedian thalami, and rostral midbrain. Chronic hypoxic-ischemic lesions were identified in the caudate nucleus and putamen bilaterally, with additional chronic ischemic changes observed in the cortex and subcortical white matter of the left parietal lobe, resulting in segmental cortical atrophy and moderate subcortical and cortical brain atrophy. Ischemic stroke was also detected in the frontotemporal, parietal, and right occipital regions, the basal ganglia on the left side, the cortex of the postcentral gyrus, the posterior superior part of the right cerebellum, middle cerebral artery territory, and acute lacunar infarct in the pons (Table 1) (Riggs et al., 1993; Sachdev et al., 2002; Temizoz et al., 2009; Rajendiran et al., 2012; Viswanathan et al., 2012; Bilir et al., 2013; An et al., 2014; Guzel et al., 2016; Kulhari et al., 2016; Bong et al., 2017; Dalugama and Gawarammana, 2018; Elavarasi et al., 2020; Ramlackhansingh and Seecheran, 2020; Karri et al., 2021; Masaraddi et al., 2021; Kabra et al., 2022; Nittner-Marszalska et al., 2022; Evcen et al., 2023; Priyadarshi et al., 2024).

Regarding angiography, MR angiographies have revealed various findings such as stenosis of the superior branch of the left middle cerebral artery, occlusion of the left internal carotid artery, absence of signal in the distal portion of the right internal carotid artery and in the middle cerebral artery, and simultaneous occlusion of the right and left internal carotid arteries. In addition, bilateral thalamic diffusion restriction was reported, which is indicative of an acute infarction in the territory of the artery of Percheron (Sachdev et al., 2002; Kulhari et al., 2016; Bong et al., 2017; Karri et al., 2021). However, one case reported normal findings in MR angiography (Kabra et al., 2022). In terms of CT angiography, a slight narrowing of the right posterior cerebral artery was reported (Nittner-Marszalska et al., 2022). As for conventional angiography, occlusion of the left internal carotid artery was reported in one case, and complete and near-complete occlusions of the right and left internal carotid arteries, respectively, were observed in a 52-year-old patient (Riggs et al., 1993; 1994).

## 4 Discussion

This systematic review aimed to consolidate existing information to evaluate the potential risk of wasp and bee stings in triggering strokes in humans. Although millions of stings from these insects occur annually worldwide, these incidents generally result in localized reactions, such as severe pain, edema, and swelling at the sting site. These symptoms typically resolve within a few hours or days and are not usually life-threatening (Gupta, 2020). In cases where the sting causes more severe reactions, symptoms often have a delayed onset, making diagnosis more challenging. For example, the time between envenomation and the onset of stroke symptoms can range from 15 min to 4 days, with a median onset time of 16 h (Moein and Zand, 2017). In most of these cases, it is more likely that a bee or a wasp will cause the negative health consequences. However, systemic toxic reactions due to the venom compounds of these insects are usually observed after 50 to 100 bee stings (Bilir et al., 2013). The occurrence of a stroke as a consequence of venom exposure is generally rare, with only a few reports in the literature until the third decade of the 21st century. Stroke development is not always linked to the number of stings, whether multiple or single, but rather depends on the individual’s idiosyncratic response, their unique physiological reactions, and any preexisting risk factors (Moein and Zand, 2017).

In this review, we observed that the available literature was limited to case reports, identifying a total of 28 reports documenting 29 cases. Notably, almost all reported cases (28 of 29) occurred in males, aligning with the higher incidence of stings in men (Table 1). According to Linard et al., 68.2% of bee stings occurred in men; similar results were found by Diniz et al., who reported that 64.2% of the 1,307 stings recorded between 2007 and 2013 occurred in males (Linard et al., 2014; Diniz et al., 2016). This could be explained by the greater inclination of men to engage in outdoor activities. Additionally, there was an almost equal distribution of cases caused by bees and wasps, preventing the association of stroke with either species specifically.

In general, insect stings from the order *Hymenoptera*, which includes bees and wasps, usually cause uncomplicated local reactions such as pain, swelling, erythema, and bleeding at the sting site. Xi et al., in their study, described the clinical manifestations of patients with severe wasp stings and toxic reactions, highlighting renal and hepatic injury, rhabdomyolysis, hypotension, pulmonary edema, and hemolysis (Xie et al., 2013; Arif and Williams, 2025). Similarly, Cavalcante et al. reported a wide range of clinical complications resulting from bee stings some similar to those caused by wasp stings, as previously described; other clinical manifestation include atrial fibrillation, pericardial effusion, pericarditis, gastrointestinal bleeding, subconjunctival hemorrhage, encephalitis, and Guillain-Barré Syndrome (Cavalcante et al., 2024). It has also been reported that both bee and wasp stings can, in rare cases, lead to serum sickness, vasculitis, thrombocytopenic purpura, and various neurological, renal, or cardiovascular diseases (Przybilla and Ruëff, 2012). In our review, we observed that the majority of patients who develop a cerebrovascular accident present with neurological manifestations, the most frequent being right hemiparesis and facial nerve palsy, followed by altered consciousness, speech and language disturbances, and aphasia. Another common symptom in this group of patients is hypertension.

**FIGURE 4:**
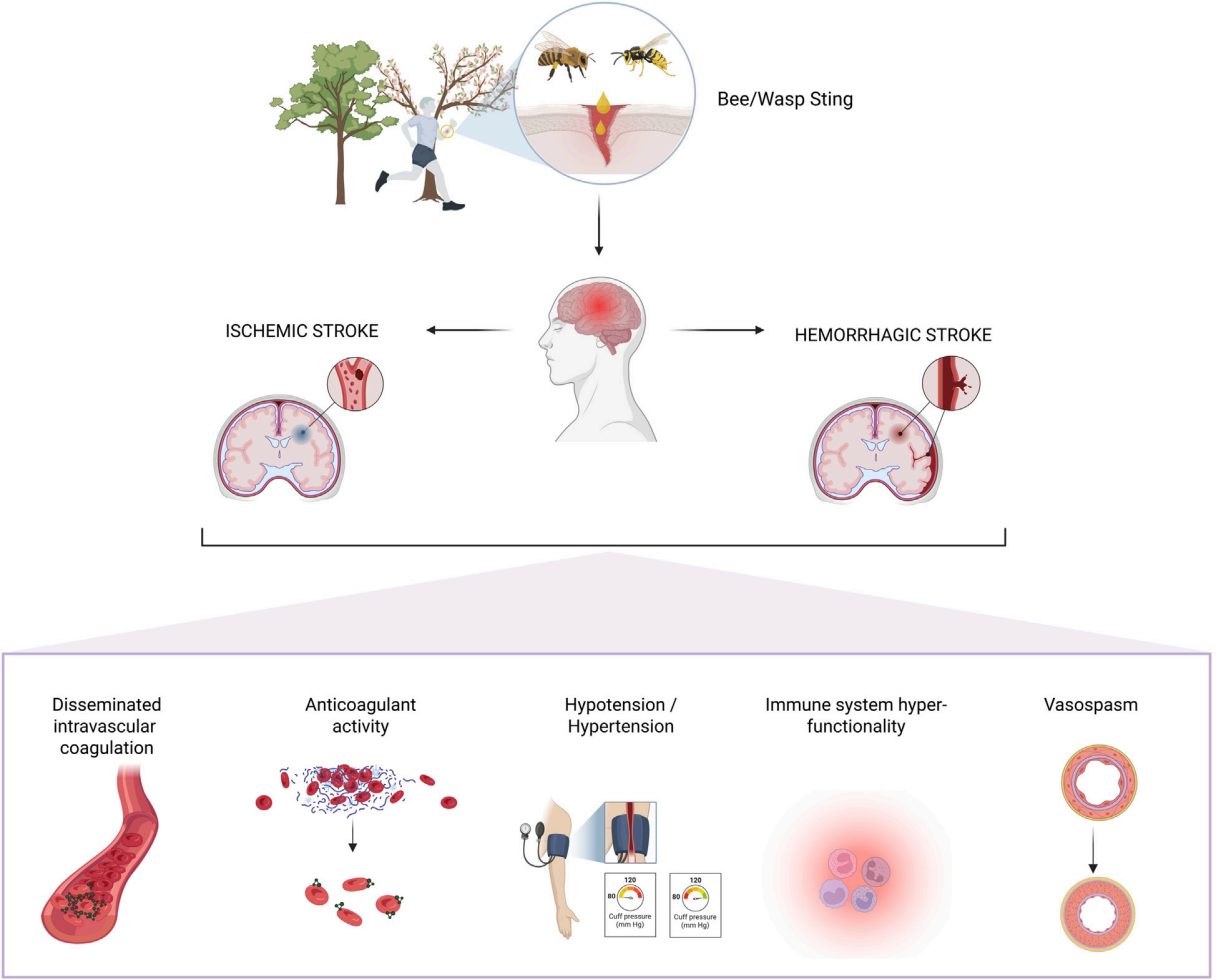
Main mechanisms associated with stroke following bee and wasp stings.

Post-mortem studies of patients who died from wasp stings reveal that the most common damage was to the nervous system, present in 70% of cases, with cerebral edema, intraventricular hemorrhage, and cerebral petechiae being the most frequent alterations (Day, 1962). The main type of stroke identified was ischemic (89.7%). However, the exact mechanism by which these stings can trigger a stroke is not fully understood. Several theories have been proposed (Figure 4). Regarding ischemic stroke, it has been suggested that bee venom toxins may cause hemolysis and endothelial damage, leading to the release of tissue thromboplastins and potentially promoting a state of disseminated intravascular coagulation (DIC), with blood vessel occlusion by fibrin thrombi (Jain et al., 2012). Additionally, bee venom contains vasoactive peptides such as thromboxane, which may cause vasoconstriction and result in ischemic stroke (Yang et al., 2022). In terms of hemorrhagic stroke, it has been described that changes in blood pressure induced by vasoactive amines released during mast cell degranulation, along with the effects of catecholamines triggered by melittin and histamine, may lead to a hypertensive state, increasing the risk of hemorrhagic stroke (Yang et al., 2022; Reddy et al., 2023). Another theory proposes that a decrease in blood pressure caused by the action of histamine and prostaglandin-2 could result in cerebral hypoperfusion and an increased risk of bleeding due to the anticoagulant activity of hyaluronidase and hemolysis mediated by phospholipase A2 (Yang et al., 2022; Reddy et al., 2023). Another proposed mechanism in the development of stroke involves hyperactivity of the immune system (Figure 4) (Yang et al., 2022).

Despite the uncertainty regarding the mechanisms of stroke production, the management of these patients should include prompt recognition and collaboration with experienced physicians to ensure the administration of specialized treatments (Jain et al., 2012). It should be noted that there are currently no protocols or clinical practice guidelines for the treatment of cerebral infarction associated with bee or wasp stings. In the case of bees, it is recommended to remove stingers embedded at the sting site as soon as possible, as they continuously inject venom (Yang et al., 2022).

In today’s world, as more people live on the edges of agricultural zones and in proximity to fields, bees and other stinging insects increasingly find themselves nesting in homes or buildings close to human activity; furthermore, climate change may be indirectly exacerbating risks related to animals and has significant impacts on venomous species (da Silva Freitas et al., 2024). This shift underscores the importance of public health awareness and a One Health approach, which recognizes the interconnectedness of human, animal, and environmental health. Proactive measures and education are crucial to prevent and manage the potential health risks posed by these insects as their interactions with humans become more frequent.

Among the limitations of this study is that it was carried out based on case reports which, due to their nature and the fact that they do not have control groups, do not allow establishing a causal relationship. Furthermore, these studies do not allow us to identify risk factors that predispose to the development of stroke after the sting of bees or wasps, as well as an average number of stings to develop it. Although most case reports do not specify the species involved, in certain instances *Vespa velutina* has been identified (Ciron et al., 2015). This invasive species, already established in Europe, Japan, and South Korea, has been linked to increased sting-related morbidity and mortality. In Europe, some regions have reported sting-related death rates as high as 2.22 per million inhabitants annually, largely attributed to *Vespa velutina* (Feás, 2021). It is also important to highlight the potential for publication bias, as the reported cases are likely to represent primarily those with severe clinical manifestations following bee or wasp stings. In contrast, cases with mild or transient symptoms may go unreported. The possibility of underreporting constitutes another significant limitation, as it hinders a comprehensive understanding of the problem. This substantially limits the ability to analyze both the clinical spectrum and the true epidemiological burden of these events. Underreporting may be partly due to the fact that many of these cases occur in low- and middle-income countries or in rural areas with limited access to healthcare services, which affects the diagnosis, management, and reporting of such cases. Finally, the presence of confounding factors cannot be ruled out, as many reports lack information on concurrent exposures, lifestyle habits such as tobacco, alcohol, or other substance use, and family history of cerebrovascular disease. These factors may independently influence or contribute to the observed risk, limiting causal interpretation.

## 5 Conclusion

Although cerebrovascular events such as ischemic and hemorrhagic strokes following bee or wasp stings are rare, scientific evidence confirms that the risk is real and should not be underestimated. The profound impact of a stroke extends far beyond the initial event, often resulting in life-altering consequences for affected individuals and placing a significant burden on healthcare systems. Given the severity of these outcomes, hospitals and healthcare centers must remain vigilant in recognizing and managing these potential complications.

Protocols should be established to ensure the timely diagnosis and treatment of these rare but serious events. Neuroimaging studies such as computed tomography (CT) or magnetic resonance imaging (MRI) should be performed in patients presenting with neurological symptoms (e.g., headache, loss of consciousness, speech disturbances, limb weakness, seizures, among others), whether these occur during initial medical evaluation, hospitalization, or after discharge. Additionally, the implementation of clinical observation protocols is essential. Observation periods ranging from 24 to 48 h may be warranted, depending on the severity of symptoms or the presence of comorbidities or risk factors that may increase the likelihood of cerebrovascular events. It is also necessary to train healthcare personnel to recognize these rare but potentially life-threatening complications and to strengthen reporting and case documentation systems to enhance epidemiological surveillance.

Moreover, considering the increasing interaction between humans and stinging insects especially in areas where urban development encroaches on natural habitats public health initiatives should emphasize awareness and preparedness. Continued research, particularly using animal models, is essential to better understand the pathophysiology of stroke following bee and wasp stings and to identify the specific species most likely to cause such complications.

## Data Availability

The original contributions presented in the study are included in the article/Supplementary Material, further inquiries can be directed to the corresponding author.
